# The role of Nox2-derived ROS in the development of cognitive impairment after sepsis

**DOI:** 10.1186/1742-2094-11-36

**Published:** 2014-02-27

**Authors:** Marina S Hernandes, Joana C D’Avila, Silvia C Trevelin, Patricia A Reis, Erika R Kinjo, Lucia R Lopes, Hugo C Castro-Faria-Neto, Fernando Q Cunha, Luiz RG Britto, Fernando A Bozza

**Affiliations:** 1 Department of Physiology and Biophysics, University of São Paulo, São Paulo, Brazil; 2 Department of Pharmacology, Institute of Biomedical Sciences, University of São Paulo, São Paulo, Brazil; 3 Laboratório de Imunofarmacologia, Instituto Oswaldo Cruz, FIOCRUZ, Rio de Janeiro, Brazil; 4 Department of Pharmacology, Faculty of Medicine of Ribeirão Preto, University of São Paulo, Ribeirão Preto, São Paulo, Brazil; 5 Instituto de Pesquisa Clínica Evandro Chagas, FIOCRUZ, e Instituto D’Or de Pesquisa e Ensino (IDOR), Rio de Janeiro, Brazil; 6 Laboratory of Cellular Neurobiology, Institute of Biomedical Sciences, University of São Paulo, Av. Professor Lineu Prestes, 1524, ZIP: 05508-900 São Paulo, Brazil; 7 Intensive Care Unit, Instituto de Pesquisa Clinica Evandro Chagas, Fundação Oswaldo Cruz, Av. Brasil, 4365, Rio de Janeiro RJ ZIP: 21040-900, Brazil

**Keywords:** Systemic inflammation, Brain, NADPH oxidase, Oxidative stress, Encephalopathy

## Abstract

**Background:**

Sepsis- associated encephalopathy (SAE) is an early and common feature of severe infections. Oxidative stress is one of the mechanisms associated with the pathophysiology of SAE. The goal of this study was to investigate the involvement of NADPH oxidase in neuroinflammation and in the long-term cognitive impairment of sepsis survivors.

**Methods:**

Sepsis was induced in WT and gp91^phox^ knockout mice (gp91^phox-/-^) by cecal ligation and puncture (CLP) to induce fecal peritonitis. We measured oxidative stress, *Nox2* and *Nox4* gene expression and neuroinflammation in the hippocampus at six hours, twenty-four hours and five days post-sepsis. Mice were also treated with apocynin, a NADPH oxidase inhibitor. Behavioral outcomes were evaluated 15 days after sepsis with the inhibitory avoidance test and the Morris water maze in control and apocynin-treated WT mice.

**Results:**

Acute oxidative damage to the hippocampus was identified by increased 4-HNE expression in parallel with an increase in *Nox2* gene expression after sepsis. Pharmacological inhibition of Nox2 with apocynin completely inhibited hippocampal oxidative stress in septic animals. Pharmacologic inhibition or the absence of Nox2 in gp91^phox-/-^ mice prevented glial cell activation, one of the central mechanisms associated with SAE. Finally, treatment with apocynin and inhibition of hippocampal oxidative stress in the acute phase of sepsis prevented the development of long-term cognitive impairment.

**Conclusions:**

Our results demonstrate that Nox2 is the main source of reactive oxygen species (ROS) involved in the oxidative damage to the hippocampus in SAE and that Nox2-derived ROS are determining factors for cognitive impairments after sepsis. These findings highlight the importance of Nox2-derived ROS as a central mechanism in the development of neuroinflammation associated with SAE.

## Introduction

The clinical observation that sepsis and systemic inflammation affect brain function is not novel [1-3]. However, it was only recently demonstrated that acute brain dysfunction is an independent cause of morbidity and mortality in septic patients [4]. The presence of acute brain dysfunction is a determining factor for long-term cognitive impairments among sepsis survivors. Clinical studies have shown that up to 60% of sepsis survivors exhibit permanent cognitive deficits and memory loss [5].

Sepsis- associated encephalopathy (SAE) and its long-term consequences in cognitive function are still poorly understood. The use of relevant preclinical models has facilitated the elucidation of central mechanisms, such as mitochondrial and vascular dysfunction, neurotransmission disturbance, inflammation and cell death [6-8]. Some studies have addressed the involvement of reactive oxygen species (ROS), reduced antioxidant capacity, and the accumulation of oxidative stress markers in experimental sepsis [9-11] and in patients [12-17]. Mitochondria are major sources of intracellular ROS and recent studies support the importance of mitochondrial ROS for immune cell function [18,19]. However, the extent to which mitochondria generate ROS *in vivo* is still controversial [20]. Another main source of ROS in the brain is NADPH oxidase.

NADPH oxidases are a family of enzymes dedicated to the generation of ROS (O2 · -) via reduction of molecular oxygen [21,22]. Members of the NADPH oxidase family include Nox1 through 5 as well as Dual oxidase 1 and 2 (Duox 1 and Duox 2). In the nervous system, the main isoforms are Nox1, Nox2 and Nox4 [23]. These isoforms have been identified in different cell types, including neurons, endothelial and glial cells [24]. Moreover, NADPH oxidase isoforms are activated by various stimuli, including damage-associated and pathogen-associated molecular patterns (DAMPs and PAMPs), inflammatory mediators and neurotoxins. NADPH oxidase dependent production of O2^·-^ has been identified as a major contributor to brain injury in cerebral ischemia [25-27], excitotoxicity [28], Alzheimer’s disease, Parkinson’s disease and other neurological diseases [29].

Here we explored the role of NADPH oxidase in different aspects of the pathophysiology of SAE. We investigated the involvement of NADPH oxidase in neuroinflammation and in the long-term cognitive impairment of sepsis survivors. Our approach included pharmacological inhibition of NADPH oxidase activity with apocynin, as well as the use of genetically deficient (knockout) mice for gp91^phox^ (gp91^phox-/-^), the catalytic subunit of Nox2. We identified Nox2 as a necessary step for glial cell activation in SAE and as a major source of oxidative stress and cognitive impairment in sepsis survivors.

## Material and methods

### Animals

Adult male C57BL/6 (wild-type (WT)) (eight to ten week-old, 25 to 30 g) were obtained from the Oswaldo Cruz Foundation (FIOCRUZ), and from the Institute of Biomedical Sciences, University of São Paulo breeding units. Gp91^phox-/-^ mice were obtained from Jackson Laboratories (Bar Harbor, ME, USA). The animals had free access to food and water and were maintained on a 12:12 hour light–dark cycle. Experiments were performed with age- and weight-matched animals. All procedures were approved by the Institutional Animal Care Committees of the Institute of Biomedical Sciences, University of São Paulo and the FIOCRUZ, Rio de Janeiro, Brazil.

### Sepsis model

Sepsis was induced by cecal ligation and puncture (CLP), a procedure that generates an acute polymicrobial peritonitis as previously described [30]. Mice were anesthetized with an intraperitoneal (ip) injection of ketamine (100 mg/kg) and xylazine (10 mg/kg) and a 1 cm incision was made on the abdomen. The cecum was exposed and ligated below the ileocecal junction. A double puncture was made using a 22-gauge needle, to induce mild sepsis. Sham-operated animals (controls) underwent an identical laparotomy but without CLP. Alternatively, experimental sepsis was induced by an ip injection of the fecal slurry, which induces acute polymicrobial peritonitis and a septic syndrome similar to CLP. Both models show similar inflammatory response profiles in terms of mortality, blood cytokines and neuroinflammation (data not shown). The intraperitoneal injection of the fecal slurry model was chosen for behavioral studies to avoid surgery and the use of anesthetics that might affect pathways involved in cognitive impairment induced by sepsis. All mice received antibiotic therapy by subcutaneous injection of ertapenem or meropenen (20 mg/kg) (Merck Research Laboratory, Whitehouse Station, NJ, USA) beginning six hours after sepsis induction and then every twelve hours up to day four. Animals received 1 ml of sterile saline subcutaneously (sc) as fluid resuscitation after sepsis induction.

### Apocynin treatment

Apocynin has been widely used as a pharmacological tool to inhibit NADPH oxidase activity [31,32]. Its mechanism of action involves blocking the translocation of the cytosolic subunit p47^phox^ to the plasma membrane, thereby inhibiting Nox2 activation [33]. Apocynin treatment has shown anti-inflammatory effects in some models of inflammatory diseases, such as rheumatoid arthritis [34], Parkinson's disease [32], ALS [35] and brain ischemia [36], as well as in our models of sepsis (data not shown). To access the involvement of Nox2-derived ROS in glial cell activation in the hippocampus, WT mice were injected with 20 mg/kg of apocynin (Sigma-Aldrich, St. Louis, MO, USA) sc 30 minutes prior to the induction of sepsis. Tissue samples were collected at six hours, twenty-four hours and five days post-sepsis for immunoblotting, immunohistochemistry and real-time PCR analysis. To test the effects of Nox2 inhibition on behavioral outcomes, sham and septic mice were treated with 5 mg/kg of apocynin sc beginning one hour after sepsis onset and at 6, 24 and 48 hours post-sepsis. Apocynin did not affect mortality or severity scores of treated mice (data not shown).

### Real-time PCR

Hippocampi from sham (n = 8) and septic (n = 8) mice were directly homogenized in 1 ml TRIzol (Invitrogen, Carlsbad, CA, USA) and total RNA was isolated following the manufacturer's suggested protocol. PCR reactions were performed, recorded, and analyzed using the Corbett Research system (Corbett Life Sciences, Sydney, Australia). The specificity of the SYBR® green assay was confirmed by melting-point analysis. Expression data were calculated from the cycle threshold (Ct) value using the ΔCt method for quantification [37]. Gene expression of GAPDH mRNA (sense: 5′-GTGCAGTGCCAGCCTCGTCC-3′; antisense: 5′- CAGGCGCCCAATACGGCCAA-3′) was used for normalization. The results were expressed as percent increases. All oligonucleotides and reagents utilized in this protocol were purchased from Invitrogen, Carlsbad, CA, USA. Sequences used were: *Nox2* (sense: 5’-TCAAGACCATTGCAAGTGAACAC-3’; antisense: 5’-TCAGGGCCACACAGGAAAA-3’) and *Nox4* (sense: 5’- TGGGCGTCCTCGGTGGAAACT-3’; antisense: 5’- TGGGTCCACAGCAGAAAACTCCA-3’).

### Measurement of oxidative stress

Oxidative stress was assessed by Western blotting for 4-hydroxynonenal, a marker of tissue oxidative stress [38]. Mice were deeply anesthetized with isoflurane; the hippocampi were collected at different time points after sepsis induction and immediately frozen in dry ice. The tissue was homogenized in ice-cold Tris–HCl buffer containing protease and phosphatase inhibitor cocktails (Complete, Roche, Indianapolis, IN, USA). Protein concentration was measured with a BCA protein assay kit (Thermo Fisher Scientific, Waltham, MA, USA). Protein electrophoresis was made in Polyacrylamide 4 to 15% gradient gels (Pre-cast, BioRad, Hercules, CA, USA) and proteins were transferred to PVDF Immobillon-FL membranes (Millipore, MA, USA). Membranes were blocked with Odyssey Blocking Buffer reagent (Li-Cor Biosciences, Lincoln, NE, USA) and incubated overnight with a mouse monoclonal antibody anti-HNE (1:500, Abcam, Cambridge, MA, USA) and a rabbit polyclonal anti-cyclophilin B (1:5,000; Pierce Biotechnology, Inc. Rockford, IL, USA); the latter was used as a loading control as described elsewhere [39]. After washing with PBS, membranes were incubated with IRDye secondary antibodies (Li-Cor Biosciences) for one hour. Immunoreactivity was visualized with an Odyssey Infrared Imaging System (Li-Cor Biosciences, Lincoln, NE, USA). This system allows multiplex detection of antigens in one membrane using primary antibodies from different species, without the need for membrane stripping.

### Immunohistochemistry

Mice were deeply anesthetized with isoflurane and subjected to transcardial perfusion with a saline solution followed by 4% paraformaldehyde (PFA) dissolved in 0.1 M PBS at pH 7.4. The brains were collected, post-fixed in PFA overnight, and transferred to a 20% sucrose solution for 48 hours to ensure cryoprotection. For glial cell morphological studies, brain sections (40 μm) were obtained in a cryostat (Leica Microsystems, Germany, model CM1850) and stored floating in PBS. Sections were incubated with a blocking buffer containing 0.3% Triton X-100, 2% normal goat serum and 1% BSA for two hours. Sections were then incubated overnight with a rabbit IgG anti-ionized calcium binding adaptor molecule 1 (1:200; Iba1; Wako Chemicals Inc., Richmond, VA, USA) or with a rabbit IgG anti-glial fibrillary acidic protein (1:1,000; GFAP; Millipore, MA, USA) diluted in blocking buffer. Following three washes with PBS, sections were incubated for two hours with a secondary antibody, Alexa 488 goat anti-rabbit IgG diluted 1:1,000 in blocking buffer. After washing out the secondary antibody, sections were mounted on slides with Vectashield Mounting Media for Fluorescence with 4',6-diamidino-2-phenylindole (DAPI) (Vector Laboratories, Burlingame, CA, USA) for nuclear staining. Confocal images were obtained with a confocal laser-scanning microscope (Leica Microsystems, Germany, model TCS SP5 AOBS) with a 63x oil immersion objective.

For quantitative studies, 30 μm brain sections were incubated free-floating for 12 to 16 hours with anti-OX42 (CD11b/c, Biosciences, San Jose, CA, USA) and anti-GFAP (Immunon, Pittsburgh, PA, USA) diluted 1:1,000 in 0.3% of Triton X-100 containing 0.05% normal goat serum. Following three washes of ten minutes each with PBS, sections were incubated for two hours with a biotinylated secondary antibody (donkey anti-mouse IgG, Jackson ImmunoResearch, West Grove, PA, USA, 1:200), followed by the avidin-biotin complex (1:100; ABC Elite kit, Vector Labs, Burlingame, CA, USA). After washing, the sections were reacted with 0.05% 3,3-diaminobenzidine and 0.01% hydrogen peroxide in PBS. Intensification was conducted with 0.05% osmium tetroxide in water. The sections were mounted on gelatinized slides, dehydrated, cleared and cover-slipped. Controls for immunostaining included the omission of the primary antibody and its substitution for normal goat serum, which completely eliminated staining. The material was analyzed using a light microscope, and digital images were semi-quantitatively analyzed using ImageJ software (National Institutes of Health, USA). OX42 and GFAP immunostaining were evaluated in terms of optical density within 0.4 mm^2^ areas for the CA1, CA3 and DG regions. The mean optical density of labeled areas was compared with the mean density of neighboring, non-labeled areas in the same sections, to obtain a labeling index reflecting the mean signal-to-noise ratio as previously described [40].

### Behavioral tests

Behavioral outcomes were evaluated 15 days after sepsis onset. The step-down inhibitory avoidance test was performed as we previously described [41]. In the training trial, animals were placed on a platform and their latency to step down on the grid with all four paws was measured with an automatic device. Immediately upon stepping down on the grid, the animals received a 0.6 mA/3.0-second foot shock. A retention test trial was performed 24 hours after training and duration on the grid was recorded (cut-off of 180 seconds). The Morris water maze has been extensively used to examine learning and memory deficits in many neurological disease models. Damage to the hippocampus causes impairment in spatial memory function in the water maze [42]. Animals were trained to find the platform for four days with 60-second trial sessions and their time to find the platform was measured. On the fifth day, a trial in the absence of the platform was run and the exploration time in the quadrant where there was previously a platform was measured and expressed as latency time.

### MCP-1 and IL-1 measurements

The inflammatory response induced by sepsis was evaluated by measuring the levels of monocyte chemoattractant protein (MCP)-1 and IL-1 in the plasma and in the peritoneal cavity using an ELISA method. Mice were killed in a carbon dioxide chamber six hours after sepsis onset, and the peritoneal cavity was opened and rinsed with 3 ml of PBS solution. The peritoneal fluid was collected for determination of cytokines levels. Blood samples were collected from a peripheral vein and kept on ice. Plasma was collected by centrifugation at 800 g for 15 minutes at 4°C, aliquoted, and stored at -70°C until the analysis day. All measurements were performed following the manufacturer’s instructions (R&D systems Duo set kit, Minneapolis, MN, USA).

### Statistical analysis

The results are presented as the mean ± standard error of the mean (SEM). Statistical analyses of data were generated using GraphPad Prism, version 3.02 (GraphPad Software Inc., San Diego, CA, USA). Statistical comparisons of more than two groups were performed using an analysis of variance (ANOVA) and were analyzed by the Mann–Whitney *U*-test or the Kruskal-Wallis test. In all cases, *P* ≤ 0.05 was considered statistically significant.

## Results

### Oxidative stress in the hippocampus is associated with *Nox2* expression

**Figure 1:**
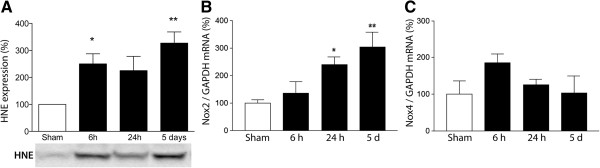
**Oxidative stress in the hippocampus is associated with *Nox2 *expression after sepsis induction.** Hippocampi from WT mice were obtained at six hours, twenty-four hours and five days after sepsis onset. **(A)** Tissue oxidative stress was assessed by measuring 4-hydroxynonenal (4-HNE) levels, expressed as the percent increase over sham. The bottom figure shows one representative Western blot with two bands per experimental condition. **(B)***Nox2* and **(C)***Nox4* mRNA expression in the hippocampus after sepsis induction. White bars represent shams and black bars represent sepsis. The statistical significance is expressed as: *P < 0.05 versus sham. n = 5 to 7 in each group. Cyc: cyclophilin B.

**Figure 2:**
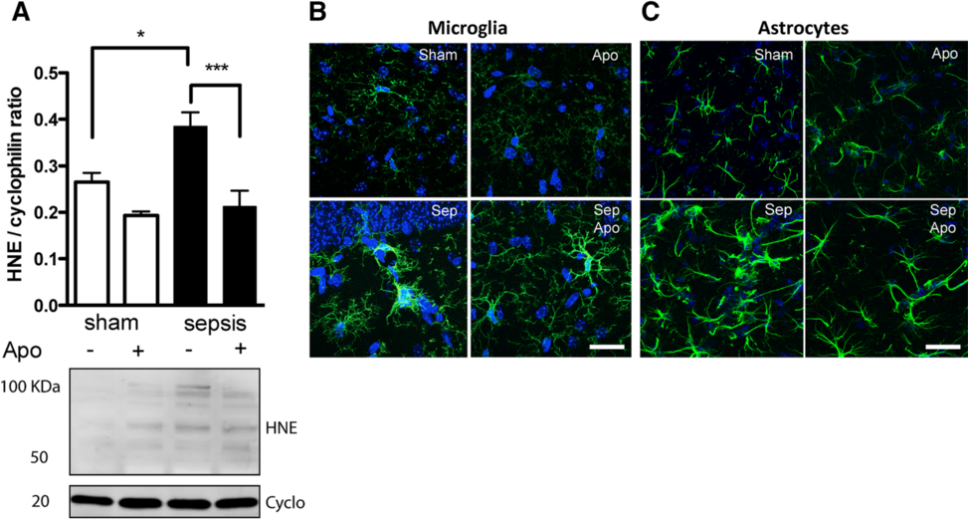
**Apocynin treatment prevents oxidative stress and astrogliosis in the hippocampus early after sepsis. (A)** WT mice were treated with apocynin one hour after sepsis onset. Brain oxidative stress was measured six hours after sepsis onset. Values are ratios of 4-HNE/cyclophilin B (a low molecular weight loading control) densitometry, expressed as mean ± SEM (n = 4 each group). The bottom figure shows two representative bands of Sham, Sham + Apo, Sepsis and Sepsis + Apo. The statistical significance is expressed as: *P < 0.05 and ***P < 0.001. **(B)** Representative digital images of Iba-1 immunoreactivity (in green) in the CA1 region indicate that apocynin treatment did not affect microglia activation. **(C)** Representative digital images of GFAP (in green) immunoreactivity in CA1 region indicate thickening of astrocyte processes, especially around blood vessels; apocynin treatment decreased astrogliosis after sepsis. Nuclear 4',6-diamidino-2-phenylindole (DAPI) staining is shown in blue. Images are representative of three independent experiments (n = 6 per experimental group). Apo: apocynin. Scale bar: 20μm.

To evaluate oxidative damage in the hippocampus of septic mice, we measured the expression of 4-hydroxynonenal (4-HNE), a by-product of lipid peroxidation. We observed a progressive increase in 4-HNE levels in the hippocampus early after sepsis that was accompanied by a significant increase in *Nox2* gene expression. 4-HNE was detected at six hours, twenty-four hours, and up to five days post-sepsis (Figure 1A). Real-time PCR analysis revealed that *Nox2* gene expression was significantly induced in the hippocampus at twenty-four hours and five days, while *Nox4* was not significantly changed, indicating that *Nox2* is associated with brain oxidative stress in SAE (Figure 1B, C). To clarify the role of Nox2 in the oxidative damage in SAE, we treated mice with apocynin. The treatment with apocynin prevented the 4-HNE increase in the hippocampus induced by sepsis (Figure 2A), indicating that Nox2 must be a major contributor to hippocampal ROS generation in SAE.

### Apocynin modulates neuroinflammation in sepsis- associated encephalopathy (SAE) by inhibiting astrogliosis

Six hours after sepsis onset there is already a significant activation of astrocytes and microglia in WT animals. Activated microglia presented increased Iba1 immunoreactivity and hypertrophic morphology (Figure 2B). Astrocytes activation presented thickening of cellular processes and increased expression of GFAP (Figure 2C). Astrocytes and microglia were affected in different ways by apocynin treatment. Astrocyte activation was inhibited by apocynin, whereas microglia activation was not affected (Figure 2).

**Figure 3:**
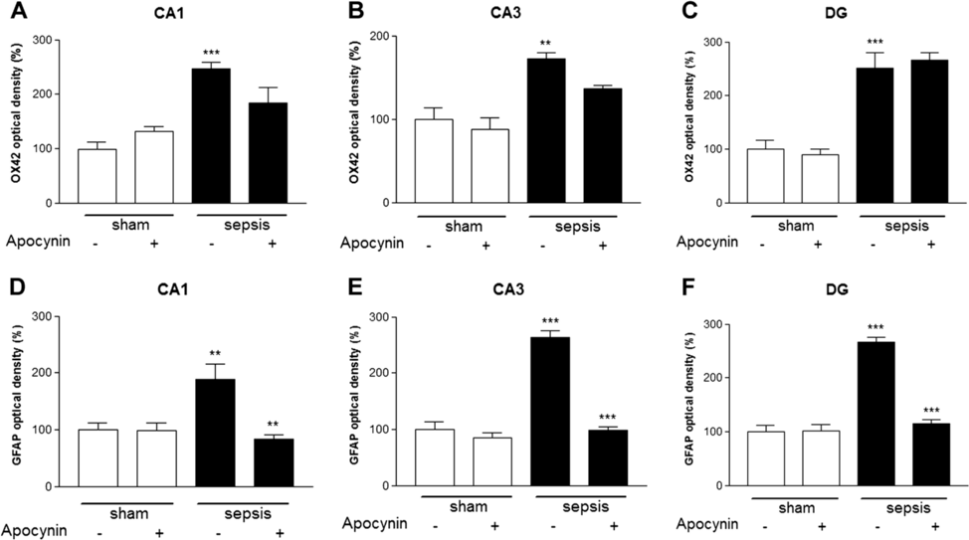
**Apocynin treatment selectively inhibits astrocyte activation after sepsis.** Effects of sepsis induction on OX42-like immunoreactivity in CA1 **(A)**, CA3 **(B)** and DG **(C)** and GFAP immunoreactivity in CA1 **(D)**, CA3 **(E)** and DG **(F)** in the hippocampi of animals allowed to survive for five days after sepsis onset. The graphs depict optical density data. In A, B and C, the statistical significance is expressed as: ***P < 0.001 and **P < 0.01 versus sham. In D, **P < 0.01 versus sham and **P < 0.01 versus sepsis. In E and F, ***P < 0.001 versus sham and ***P < 0.001 versus sepsis. n = 5 to 6 in each case. DG: dentate gyrus.

**Figure 4:**
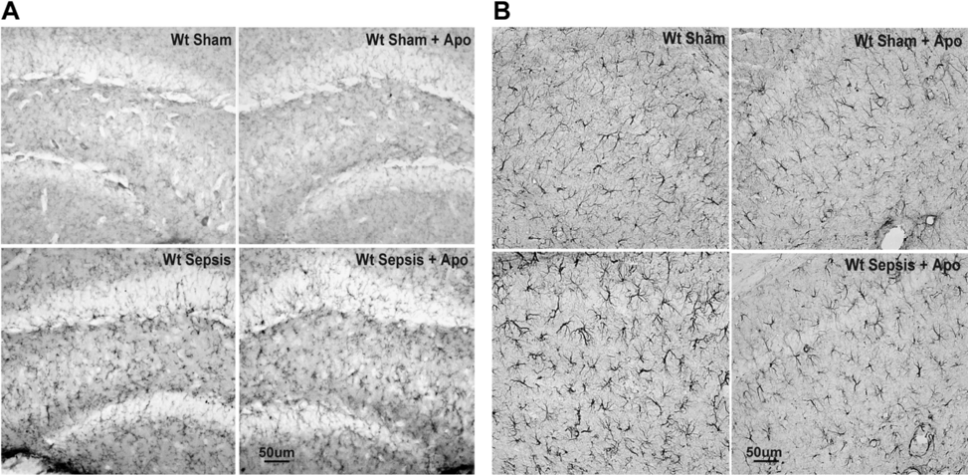
**Apocynin inhibits astrogliosis in the dentate gyrus of the hippocampus.** Representative digital images of OX42-like immunoreactivity **(A)** and GFAP-like immunoreactivity **(B)** in the dentate gyrus (DG) of the WT Sham, WT Sham apocynin, WT Sepsis, WT Sepsis apocynin groups analyzed five days after sepsis onset.

Microglia and astrocyte activation were more pronounced five days after sepsis onset, so we performed quantification of glial cells activation in different regions of the hippocampus at this time point to confirm the morphological findings. Microglial activation was more prominent within the CA1 and DG regions (CA1 *circa* 146%; CA3 *circa* 73%; and DG *circa* 151%). Apocynin treatment was not able to prevent microglial activation induced by sepsis in CA1, CA3 and DG (Figures 3A, B, C and 4A). Septic mice exhibited a significant increase in the number of activated astrocytes with highly ramified and thick processes (CA1 *circa* 87%; CA3 *circa* 163%; and DG *circa* 166% versus control). The treatment with apocynin reversed astrocyte activation in all of the hippocampal regions analyzed (CA1 *circa* 56%; CA3 *circa* 63%; and DG *circa* 56%, Figures 3D, E, F and 4B).

### Neuroinflammation induced by sepsis- associated encephalopathy (SAE) is impaired in Nox2 knockout mice

**Figure 5:**
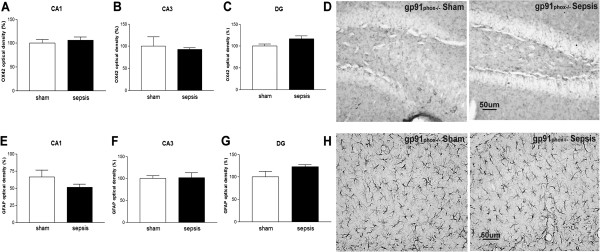
**Absence of Nox2 prevents glial cell activation in gp91**^**phox-/-**^** mice.** Effects of sepsis induction on OX42-like immunoreactivity in CA1 **(A)**, CA3 **(B)** and DG **(C)** and GFAP immunoreactivity in CA1 **(E)**, CA3 **(F)** and DG **(G)** of gp91^phox -/-^ mice in the hippocampi of animals allowed to survive for five days after sepsis onset. The graphs show optical density data. n = 5 to 6 in each case. **(D)** Representative digital images of OX42-like immunoreactivity into dentate gyrus (DG) in gp91^phox-/-^ sham and gp91^phox-/-^ sepsis mice. **(H)** Representative digital images of GFAP-like immunoreactivity into DG in gp91^phox-/-^ sham and gp91^phox-/-^ sepsis mice.

To further investigate the role of Nox2 in glial cell activation, we used gp91^phox-/-^ mice and evaluated astrocyte and microglia activation in different regions of the hippocampus five days after sepsis. We observed no discernible differences in CD11b or GFAP immunostaining between sham gp91^phox-/-^ and septic gp91^phox-/-^ mice (Figure 5). These results indicate that, although apocynin treatment did not prevent microglia activation, a functional Nox2 was necessary for microglia activation in SAE. Astrocyte activation induced by sepsis was also impaired in gp91^phox-/-^ mice, indicating a complete dependence of astrocytes on Nox2 in order to become activated (Figure 5).

### Acute apocynin treatment prevents long-term cognitive impairment after sepsis

**Figure 6:**
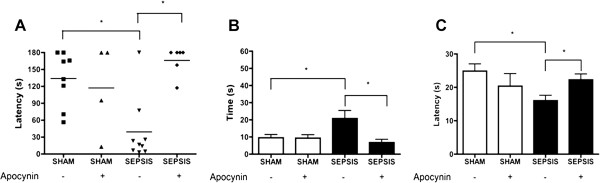
**Apocynin treatment prevents cognitive impairment after sepsis.** Septic encephalopathy was induced in WT mice (n = 5 to 10/group) via intraperitoneal inoculation with fecal slurry. The control group was injected with saline (0.9%). Mice were divided into two groups and treated with apocynin (5 mg/kg body weight) one hour after feces injection and every 24 hours for 3 days. All the animals received imipenem (10 mg/kg body weight) six hours after infection and every 24 hours for 3 days. **(A)** Step-down inhibitory avoidance test. Aversive memory was tested 24 hours after training by recording the latency time on the platform (with a cut-off of 180 seconds). Data are expressed as individual values and horizontal lines represent the mean latency in seconds; significant differences were found between sham versus septic mice (Mann–Whitney *U*-test, *P < 0.05). Spatial memory impairment was evaluated with the Morris water maze. **(B)** Time to find the platform. Student *t*-test, *P < 0.05. **(C)** Trial in the absence of platform. Latency represents the exploration on the quadrant where the platform was before removal. Student *t*-test, *P < 0.05.

To test the hypothesis that Nox-derived oxidative stress determines behavioral outcomes in sepsis survivors, we treated animals with low doses of apocynin (5 mg/kg) throughout the acute phase of sepsis and performed behavioral tests to evaluate cognitive function in survivors fifteen days after sepsis onset. Mice that survived sepsis presented significantly lower latency time on the inhibitory avoidance test, but septic mice treated with apocynin did not show any memory deficits in this test (Figure 6A). In the Morris water maze test, sepsis survivors presented an increase in the time to find the platform (Figure 6B) and spent less time in the right quadrant (Figure 6C), whereas sham animals and apocynin-treated mice all had better performances in both tests (Figure 6). These results indicate that Nox2-derived oxidative damage to the hippocampus is an important factor implicated in the long-term cognitive impairment associated with sepsis.

### Systemic inflammation induced by sepsis is not decreased by the pharmacological inhibition of Nox2

**Figure 7:**
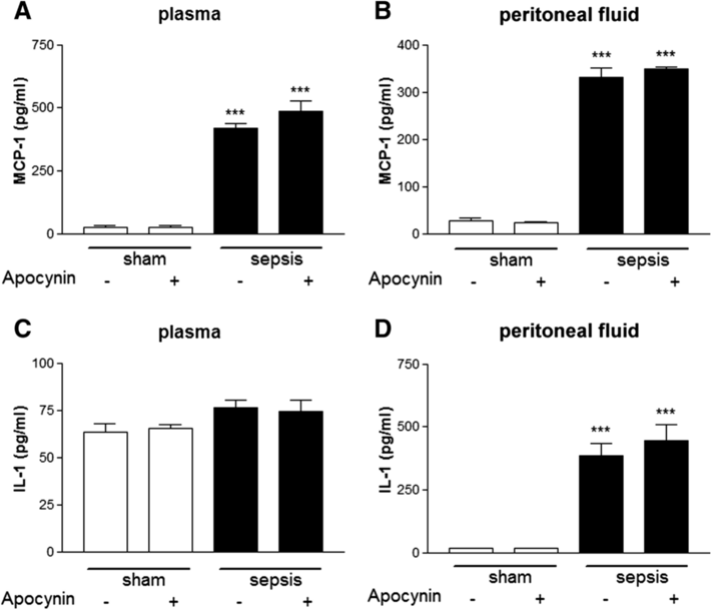
**Systemic inflammation induced by sepsis is not affected by the pharmacological inhibition of Nox2.** Effects of sepsis induction on MCP-1 and IL-1 levels in the plasma and peritoneal fluid of WT animals treated with apocynin. Mice were allowed to survive for six hours after sepsis onset. All concentrations were expressed in pg/ml. The statistical significance is expressed as: ***P < 0.001; n = 6 to 10 for each experimental group tested.

In order to investigate whether the protective effects observed by the pharmacological inhibition of Nox2 on oxidative stress, neuroinflammation and in the development of long-term cognitive impairment were secondary to a decreased systemic inflammation, animals were treated with apocynin and cytokines levels were measured after sepsis induction. We specifically investigated the ability of apocynin to decrease MCP-1 and IL-1 levels in plasma and in peritoneal fluid six hours after sepsis onset. As shown in Figure 7A and B, we observed a significant increase in MCP-1 levels in both plasma and in peritoneal fluid after sepsis onset, which was not inhibited by apocynin treatment. IL-1 levels were found significantly increased only in peritoneal fluid (Figures 7C and D). Similarly, apocynin treatment was not able to significantly inhibit IL-1 increased levels. These results suggest that the apocynin treatment was not able to prevent systemic inflammation induced by sepsis as opposed as its central effects.

## Discussion

The results presented in this study provide evidence that Nox2 is the main source of ROS involved in the oxidative damage to the hippocampus in SAE and that Nox2-derived ROS are determining factors for cognitive impairments after sepsis. There are numerous experimental findings that support these conclusions. First, there is a progressive oxidative damage to the hippocampus, identified by increased 4-HNE expression, associated with an increase in *Nox2* gene expression in the first days after sepsis. Second, pharmacological inhibition of Nox2 with apocynin completely inhibits hippocampal oxidative damage in septic animals. Third, pharmacological inhibition or the absence of Nox2 in gp91^phox-/-^ mice prevents glial cell activation, one of the central mechanisms associated with SAE and other neurodegenerative diseases [43-45]. Finally, treatment with apocynin in the acute phase of sepsis prevents the development of long-term cognitive impairment in the survivors. Our results confirm previous findings that oxidative damage to the hippocampus is involved in the development of cognitive impairment in SAE. These results also highlight the importance of Nox2-derived ROS as a central mechanism in glial cells activation and identify Nox2 as a potential target for future therapies to prevent SAE.

Sepsis-induced organ dysfunction has been attributed to a tissue adaptation to systemic inflammation, which involves mitochondrial dysfunction, oxidative stress and bioenergetics impairments [46,47]. Evidence from the literature supports the hypothesis that alterations in mitochondrial function may increase O2^·-^ generation as a byproduct of the electron transport chain, especially O2^·-^ derived from complex I [48-50]. However, the role of complex I as the major site of ROS production in intact mitochondria in the absence of respiratory chain inhibitors remain controversial. Previous studies from our group demonstrated a reduction in oxidative phosphorylation efficiency and complex IV activity in brain tissue 24 hours after sepsis onset in the CLP mouse model. In this study, we were not able to detect mitochondrial ROS production [47], suggesting that other pathways could be responsible for the oxidative stress observed in the brain tissue of septic rodents [9].

Increased NADPH oxidase activity has been implicated in a variety of neurodegenerative conditions, such as Parkinson’s and Alzheimer’s diseases (reviewed by [23,29]). There is increasing evidence that aldehyde molecules generated endogenously during the process of lipid peroxidation, such as 4-HNE, are causally involved in most of the pathophysiological effects associated with oxidative stress *in vivo*[38]. Therefore, we hypothesized that ROS derived from NADPH oxidase is the main cause of oxidative stress and brain dysfunction after sepsis. To test this hypothesis, we used pharmacological inhibition of NADPH oxidase and genetic deletion of the catalytic subunit gp91^phox^ to evaluate the acute and long-term outcomes of sepsis. Our data show that 4-HNE levels are increased in the brains of septic mice, beginning six hours and up to five days post-sepsis. Real-time PCR analysis demonstrated that *Nox2*, but not *Nox4* mRNA, was induced in the hippocampus 24 hours after sepsis, whereas oxidative stress was detected earlier at six hours, suggesting that NADPH oxidase activity is induced before the increase in gene expression. Treatment with apocynin inhibited the oxidative stress at six hours, indicating that it might be caused by an increase in Nox2 enzyme activity. Apocynin reacts with the cysteine residues of p47^phox^, a cytosolic component of the complex, inhibiting its association with the Nox2-p22^phox^ heterodimer and, consequently, its enzyme activity. The early increase in NADPH oxidase activity followed by an increase in gene expression has been demonstrated in other models of acute brain injury [51]. Thus, our data add to previous observations that mitochondria are not the main source of ROS in the nervous system after sepsis induction, and a significant portion of ROS production in the hippocampus under septic conditions is attributable to Nox2.

Nox2 has been shown to regulate intracellular ROS levels in microglia and to result in both amplification of pro-inflammatory cytokines production and priming of microglia to additional stimuli [52]. *In vitro* studies suggested that the production of ROS from Nox2 is also essential for the morphological activation of microglia in both 6-hydroxidopamine and lipopolysaccharide (LPS)-Parkinson’s disease models [53-55]. Our immunohistochemistry data show the absence of microglia activation in gp91^phox-/-^ after sepsis induction, consistent with the essential role of Nox2 in signaling microglia activation. Nevertheless, microglial activation was not prevented by the NADPH oxidase inhibitor apocynin. One possibility to explain this finding is that apocynin treatment does not inhibit microglia activation, but alters its phenotype to an anti-inflammatory M2-like phenotype, as recently demonstrated by Choi and colleagues [56].

In contrast, astrocyte activation after sepsis was significantly inhibited by a single dose of apocynin, suggesting that Nox2-derived ROS played a role in the signaling events leading to astrocyte activation after sepsis. Similarly, it has been shown that ischemic animals treated with apocynin showed significantly less reactive astrocytes compared to the non-treated group [57]. NADPH oxidase activation in astrocytes may lead to oxidative stress, glutathione depletion, and neuronal death [58,59].

Patients with chronic granulomatous disease (CGD), an immunodeficiency syndrome caused by disabling mutations of genes encoding the NADPH oxidase subunits [60,61], have an increased susceptibility to fungal and bacterial infections. Pre-clinical models using NADPH oxidase-deficient mice [62,63] have shown intrinsic defects in the control of inflammation. Han *et al*. (2013) demonstrated that p47^phox-/-^ mice have increased lung inflammation after LPS instillation and that this effect is due to increased NF-κB binding to DNA [64]. Therefore, inhibition of NADPH oxidase may be protective to the central nervous system, but may have deleterious effects in the control of systemic inflammation and/or infection. It is important to note that this does not seem to be the case in our study, because we did not observe increased mortality in apocynin-treated animals (data not shown) and in the inflammatory response induced by sepsis (as demonstrated by the evaluation of proinflammatory cytokines levels); this outcome may be explained by the low doses used to block NAPDH oxidase in our conditions.

**Figure 8:**
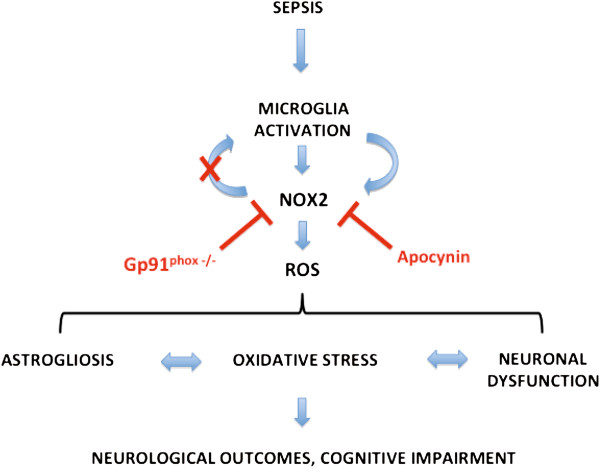
**Summary of the main findings.** This figure highlights the involvement of Nox2 in brain dysfunction associated to sepsis- associated encephalopathy (SAE) and the long-term consequences. Nox2 activation is inhibited by apocynin, preventing ROS production without affecting microglial activation. Gp91^phox^ gene deletion impairs microglial activation after sepsis.

Although there is enough evidence from the literature that sepsis survivors develop cognitive impairments, the molecular mechanisms of cognitive impairment in sepsis are far from understood. Several hypotheses were created to explain the brain dysfunction associated with sepsis, including neuroinflammation, neurotransmitter disturbances, apoptosis, and oxidative stress. Evidence from animal studies links oxidative damage and long-term cognitive impairment. Barichello *et al*. (2007) demonstrated that the administration of a combination of antioxidants, N-acetylcysteine plus deferoxamin, in the acute phase of sepsis prevented long-term oxidative damage [65]. In our study, we demonstrated that Nox2-derived ROS are determinants of neuroinflammation and of the development of long-term cognitive impairment. Strategies to modulate Nox2 activity may be useful in the future to prevent SAE development (Figure 8).

## Conclusions

The results of the present study demonstrate that Nox2 is essential for glial cell activation and emphasize the critical role of oxidative damage and Nox2-derived ROS as central factors contributing to acute and long-term brain dysfunction after sepsis.

## Abbreviations

GFAP: glial fibrilary acidic proteins; HNE: hydroxynonenal; Iba-1: ionized calcium binding adaptor molecule 1; LPS: lipopolysaccharide; NADPH: nicotinamide adenine dinucleotide phosphate; ROS: reactive oxygen species; SAE: sepsis- associated encephalopathy.

## Competing interests

The authors declare that they have no competing interests.

## Authors’ contributions

MSH PhD: performed histological assessments, data analysis, and drafted the manuscript. JCd’A PhD: performed histological and biochemical assessments, data analysis, and final manuscript preparation. SCT MSc: performed animal surgeries and data analysis. PAR PhD: performed and analyzed the behavioral studies. ERK PhD: performed experiments. LRL PhD: supervised overall project. HCC-F-N MD, PhD: supervised behavioral studies and assisted in manuscript preparation. FQC PhD: supervised animal surgeries and designed studies. LRGB PhD: designed studies, supervised histological assessments and assisted in manuscript preparation. FAB MD PhD: designed studies, supervised overall project and final manuscript preparation. All authors have read and approved the final version of the manuscript.
